# Genetic variants of the vitamin K dependent coagulation system and intraventricular hemorrhage in preterm infants

**DOI:** 10.1186/1471-2431-14-219

**Published:** 2014-09-01

**Authors:** Christine Schreiner, Sévérine Suter, Matthias Watzka, Hans-Jörg Hertfelder, Felix Schreiner, Johannes Oldenburg, Peter Bartmann, Axel Heep

**Affiliations:** 1 Department of Neonatology, University of Bonn, Adenauerallee 119, Bonn 53229, Germany; 2 Institute of Experimental Hematology and Transfusion Medicine, University of Bonn, Sigmund-Freud-Strasse 25, Bonn 53127, Germany; 3 Pediatric Endocrinology Division, University of Bonn, Adenauerallee 119, Bonn 53229, Germany; 4 School of Clinical Sciences, University of Bristol, Neonatal Intensive Care Unit, Southmead Road, Bristol BS10 NB5, UK

**Keywords:** Preterm infant, Intraventricular hemorrhage, Vitamin K dependent coagulation system, Genotype

## Abstract

**Background:**

Pathogenesis of intraventricular hemorrhage (IVH) in premature infants is multifactorial. Little is known about the impact of genetic variants in the vitamin K-dependent coagulation system on the development of IVH.

**Methods:**

Polymorphisms in the genes encoding vitamin K epoxide reductase complex 1 (*VKORC1* -1639G>A) and coagulation factor 7 (*F7* -323Ins10) were examined prospectively in 90 preterm infants <32 weeks gestational age with respect to coagulation profile and IVH risk.

**Results:**

*F7*-323Ins10 was associated with lower factor VII levels, but not with individual IVH risk. In *VKORC1*-wildtype infants, logistic regression analysis revealed a higher IVH risk compared to carriers of the -1639A allele. Levels of the vitamin K-dependent coagulation parameters assessed in the first hour after birth did not differ between *VKORC1*-wildtype infants and those carrying -1639A alleles.

**Conclusions:**

Our data support the assumption that genetic variants in the vitamin K-dependent coagulation system influence the coagulation profile and the IVH risk in preterm infants. Further studies focussing on short-term changes in vitamin K-kinetics and the coagulation profile during the first days of life are required to further understand a possible link between development of IVH and genetic variants affecting the vitamin K-metabolism.

## Background

Intraventricular hemorrhage (IVH) is a serious complication in very low birth weight (VLBW) infants and is strongly associated with neuro-developmental deficits and long-term disability in the surviving infants [1]. A large study on 450 twin-pairs estimated the contribution of genetic and shared environmental factors to the risk of developing IVH at 41% [2]. Considering the immaturity of the preterm infants’ coagulation system, genetic variants known to be associated with alterations in the coagulation profile in adults have been subject to various association studies in preterm infant cohorts. However, most of them focused on prothrombotic mutations, of which the factor V Leiden mutation and the prothrombin G20210A variant were linked to the individual risk to develop IVH [3-6]. On the other hand, studies analyzing variants affecting the vitamin K dependent coagulation system in preterm infant cohorts are scarce [7].

Vitamin K is an essential cofactor in the posttranslational carboxylation converting intracellular precursors of vitamin K dependent coagulation factors to their active forms. During this carboxylation, the active coenzyme form of vitamin K, hydroquinone, is oxidized to vitamin K epoxide. Subsequently, the vitamin K epoxide reductase (VKORC1) reduces the epoxide form back to the active hydroquinone [8]. The promoter region of the *VKORC1* gene harbours a single nucleotide polymorphism (SNP) -1639G>A, which has been associated with a reduction in VKORC1 enzyme expression of up to 50% [9]. This SNP also influences the pharmacodynamics of oral anticoagulants, which act via inhibition of VKORC1, and has been reported to explain 27% of warfarin dosing variability [10].

Another functional relevant polymorphism has been detected in the gene of the vitamin K dependent clotting factor VII. The insertion of a decanucleotide at position -323 in the promotor region is associated with reduced promotor activity and decreased factor VII levels in vitro and in vivo [11,12]. Ito et al. analyzed the coagulation profile of 200 Japanese children at the age of one month and demonstrated 25% lower coagulation ability in carriers of the insertion variant [13].

The aim of our study was to determine the impact of the polymorphism *VKORC1*-1639G>A and F7-323Ins10 on the coagulation profile and IVH risk in preterm infants less than 32 weeks of gestational age.

## Methods

### Study cohort and clinical definitions

Between May 2008 and February 2010, 124 preterm infants with a gestational age of less than 32 weeks of gestational age were born at the perinatal center of the University Hospital of Bonn. 34 infants were not included based on the following criteria: congenital malformation, chromosomal abnormalities, cholestatic liver disease, virus hepatitis, congenital heart defects, and prenatal diagnosis of intraventricular hemorrhage and periventricular leucomalacia. The final study population comprised 90 infants. By reviewing birth protocols and medical charts of the patients, we collected relevant clinical data including gender, gestational age, auxological birth parameters, mode of delivery, prenatal steroids, Apgar-Score, Clinical Risk Index for Babies (CRIB)-Score and mean arterial blood pressure at admission to our neonatal intensive care unit (NICU). Small for gestational age (SGA) was defined as birth weight and/or length below the 3^rd^ centile. Gestational age was determined by first trimester ultrasound examination. Clinical chorioamnionitis was defined by CRP value in the maternal serum of >20 mg/l, maternal leukocyte count >15,000/μl and fever >38°C on the last three days before delivery.

Respiratory support via continuous positive airway pressure (CPAP) as well as intubation and mechanical ventilation were categorized positive when required within the first five days. The parameter surfactant included different modes of surfactant application within the first day of life via either endotracheal tube, Insure Sequence (*in*tubate, *sur*factant, *e*xtubate) or via gastric tube placed intratracheally in infants receiving respiratory support by CPAP. Catecholamine treatment of arterial hypotension (categorized positive when needed within the first 72 hours) followed a standardized protocol. In addition to the incidence of a patent ductus arteriosus, we separately assessed administration of indomethacin or ibuprofen and surgical closure. All clinical data were anonymized prior to statistical analyses.

The diagnosis of intraventricular hemorrhage was based on serial ultrasound examinations (8.5-10 MHz transducer, Vingmed Vivid FiVe and Philips HD7 respectively) on days 1, 3 and 7 postnatal age. The maximal grade of IVH was confirmed by cranial ultrasound on day 7 postnatal age. IVH grade I was defined as bleeding into the germinal matrix, grade II as blood within the ventricular system filling less than 50% of the ventricular volume and without distension, grade III as blood in the ventricular system with distension or dilatation, and grade IV as bleeding with parenchymal involvement [14].

### Coagulation profiles

Citrate blood samples for coagulation profile analyses were collected together with the routine laboratory testing for blood count, C-reactive protein (detection limit 0.2 mg/l) and interleukin 6 within the first hour of life prior to vitamin K administration (0.2 mg phytomenadione solution intravenously). Determined components of the coagulation profile included activities of clottable fibrinogen, activity of coagulation factors II, V, VII, VIII, and X, as well as antithrombin. Coagulation parameters were analyzed in the platelet poor plasma after centrifugation, the remaining sediment was used for DNA extraction. Details on blood sampling and laboratory methods used to determine concentrations and activities of the above-mentioned coagulation parameters are described elsewhere [15].

### Genotyping

In order to minimize the required total blood volume taken for study purposes, DNA for genotyping was extracted from remaining citrate blood sediments using a commercially available kit (Invitrogen, Carlsbad, USA). Genotypes for the polymophisms *VKORC1* -1639G>A (rs9923231) and *factor 7* -323Ins10 (rs36208070) were determined by PCR-restriction fragment length polymorphism analysis. PCR primer sequences and reaction conditions are available on request. Genotyping was successful in 87 of 90 samples for the *VKORC1*-polymorphism and in 86 samples for *F7* -323Ins-polymorphisms. 250 healthy adult blood donors, who were previously genotyped at the Institute of Experimental Hematology and Transfusion Medicine, University of Bonn, served as reference population [16].

### Statistical analysis

Data were analyzed using the SPSS version 21.0 (SPSS Inc., Chicago, IL, USA). A p-value <0.05 was considered statistically relevant. To compare values between genotype groups and between infants with and without IVH, we used Mann–Whitney U- and Fisher’s exact tests. Logistic regression analysis was performed to identify independent risk factors for the development of IVH. Because of the small number of samples homozygous for the variant allele of both polymorphisms, those heterozygous and homozygous for the variant allele were grouped for statistical analysis.

### Ethics

Written informed consent was obtained from all parents. The study was approved by the Ethics committee of the Medical Faculty of the University of Bonn (048/08).

## Results

90 preterm infants with a gestational age of less than 32 weeks born in the perinatal center of the University Hospital of Bonn between May 2008 and February 2010 were included in this prospective cohort study. IVH occurred in 17 infants (18.9%). Clinical characteristics and routine laboratory parameters of the study population are summarized in Table 1. Infants who developed an IVH were born at a significantly lower gestational age, were more frequently intubated and mechanically ventilated, and received less frequently respiratory support by CPAP compared to those without IVH. More also required surgical closure of patent ductus arteriosus. Comparison of laboratory parameters revealed significantly lower levels of the vitamin K-dependent coagulation factors II and X in the IVH group (Table 1). Clinical data stratified for *F7*-323Ins10 and *VKORC1* -1639G>A polymorphisms are presented in Tables 2 and 3.

**Table 1:** **Neonatal data and routine laboratory at the first day of life according to the diagnosis of IVH confirmed by ultrasound at the 7**^**th **^**day of life (median with range and percentages, respectively)**

	**Total (n = 90)**	**Non-IVH (n = 73)**	**IVH (n = 17)**	**p-value**
Gestational age [weeks + days]	28 + 0 (23 + 3 -31 + 5)	28 + 3 (24 + 0 - 31 + 5)	27 + 0 (23 + 3 - 30 + 2)	**0.016**
Birth weight [g]	990 (320 – 2270)	990 (400 – 2270)	920 (320 – 1495)	0.248
Male [%]	67.8	67.1	70.6	1.000
SGA [%]	18.9	19.2	17.6	0.727
Prenatal steroids [%]	67.4	68.1	64.7	0.781
AIS [%]	5.6	5.5	5.9	1.000
Cesarean section [%]	98.9	98.6	100	1.000
Surfactant [%]	83.3	79.5	100	0.065
RDS [%]	85.6	84.9	88.2	1.000
RDS [median grade]	2	2	2	0.549
CPAP [%]	88.9	93.2	70.6	**0.019**
Intubation [%]	41.1	34.2	70.6	**0.012**
PDA [%]	75.6	71.2	94.1	0.061
PDA medicament [%]	78.9	75.3	94.1	0.108
PDA OP [%]	5.6	2.7	17.6	**0.045**
Sepsis [%]	10.0	6.8	23.5	0.061
Catecholamines [%]	42.2	38.4	58.8	0.173
umbilical artery pH	7.33 (7.15 - 7.52)	7.33 (7.15 - 7.52)	7.34 (7.22 - 7.38)	0.530
Apgar 5 min	8 (2–10)	8 (2–10)	8 (6–9)	0.183
CRIB-Score	2.5 (0–16)	2.0 (0–14)	5.0 (1–16)	0.495
Mean arterial pressure [mmHg]	33.5 (19–60)	34.0 (20–60)	27 (19–42)	0.053
Hematocrit [%]	45.0 (10.7 - 59.0)	47.0 (10.7 - 59.0)	41.0 (24.0 - 59.0)	0.075
Leucocytes [×10^3^/μl]	7.24 (1.08 - 32.00)	7.39 (1.08 - 32.00)	6.63 (1.16 - 26.25)	0.613
Thrombocytes [×10^3^/μl]	160 (15–291)	150 (15–272)	175 (30–291)	0.270
Il-6 [pg/ml]	51.6 (2.0 - 689,002.0)	42.3 (2.0 - 57,050.0)	92.1 (12.9 - 689,002.0)	0.197
CRP [mg/l]	0.2 (0.2 - 78.2)	0.2 (0.2 - 78.2)	0.2 (0.2 – 20.9)	0.461
FII [activity in%]	32 (17–65)	35 (17–65)	27 (18–36)	0.001/*0.006
FVII [activity in%]	31 (7–93)	31,5 (7–93)	29 (10–48)	0.116/*0.281
FX [activity in%]	41 (14–89)	46 (14–89)	33 (24–62)	0.007/*0.023
ATIII [activity in%]	30 (13–51)	30.0 (13–51)	28 (13–45)	0.241/*0.383

*adjusted for gestational age.

Definitions: Antenatal steroids: maternal Betamethason treatment 2x 12 mg (i.v.) > 24 prior delivery; AIS: Chorioamnionitis: clinical diagnosis of chorioamnitis (maternal temperature > 38° Celsius, leucocytosis > 15,0000, CRP > 20 mg/l); SGA: birth weight < 3^rd^ centile; Catecholamine treatment (n = 130): any catecholamine treatment (Dopamine, Dobutamine, Epinephrine) to maintain blood pressure > 10^th^ centile < 72 hours postnatal age.

Bold numbers are used for results reaching statistical significance.

**Table 2:** **Neonatal data and routine laboratory at the first day of life according to *F7*-genotype (median with range and percentages, respectively)**

	***F7*-323Ins10 wildtype (wt/wt) (n = 68)**	***F7*-323Ins10 carrier (wt/10 + 10/10) (n = 18 + 0)**	**p-value**
Gestational age [weeks + days]	27 + 6 (23 + 3 - 31 + 5)	28 + 5 (25 + 2 - 30 + 6)	0.656
Birth weight [g]	990 (320–2005)	990 (405–2270)	0.255
Male [%]	66.2	73.7	0.593
IVH [%]	18.3	21.1	0.750/*0.649
Prenatal steroids [%]	65.7	73.7	0.591
Surfactant [%]	81.7	89.5	0.729
CPAP [%]	87.3	94.7	0.682
Intubation [%]	40.8	42.1	1.000
PDA medicament [%]	77.5	84.2	0.753
PDA OP [%]	7.0	0.0	0.580
Sepsis [%]	11.3	5.3	0.678
Apgar 5 min	8 (5–10)	8 (2–9)	0.444
CRIB-Score	4 (0–16)	1.5 (1–8)	0.108
Il-6 [pg/ml]	42.3 (2.0 - 689,002.0)	102.0 (10.5 - 5140.0)	0.326
CRP [mg/l]	0.2 (0.2 - 78.2)	0.2 (0.2 - 35.0)	0.917
Hematocrit [%]	46.0 (10.7 - 59.0)	43.0 (31.0 - 59.0)	0.452
Leucocytes [×10^3^/μl]	7.08 (1.08 - 32.00)	7.70 (3.29 - 26.25)	0.353
Thrombocytes [×10^3^/μl]	157 (15–278)	170 (75–291)	0.174
FII [activity in%]	34 (17–65)	29 (23–45)	0.236/*0.107
FVII [activity in%]	34 (7–93)	29 (12–48)	0.052/*0.028
FX [activity in%]	45 (23–89)	33 (14–58)	0.340/*0.013
ATIII [activity in%]	30 (13–51)	29.5 (16–48)	0.903/*0.799

*adjusted for gestational age.

Bold numbers are used for results reaching statistical significance.

**Table 3:** **Neonatal data and routine laboratory at the first day of life according to the *VKORC1*-genotype (median with range and percentages, respectively)**

	***VKORC1*-1639G>A wildtype (GG) (n = 34)**	***VKORC1*-1639G>A carrier (GA + AA) (n = 40 + 13)**	**p-value**
Gestational age	28 + 6 (24 + 0 – 31 + 5)	27 + 4 (23 + 3 – 31 + 1)	0.057
Birth weight [g]	1037.5 (410 – 2005)	915 (320 – 2270)	0.095
Male [%]	67.6	69.8	1.000
IVH [%]	26.5	13.2	0.158/*0.019
Prenatal steroids [%]	64.7	69.2	0.814
Surfactant [%]	82.4	83.0	1.000
CPAP [%]	94.1	86.8	0.473
Intubation [%]	44.1	39.6	0.824
PDA medicament [%]	79.2	76.5	0.795
PDA OP [%]	5.9	5.7	1.000
Sepsis [%]	11.8	7.5	0.706
Apgar 5 min	8 (2 – 9)	8 (5 – 10)	0.062
CRIB-Score	2 (0 – 14)	3.5 (0 – 16)	0.166
Il-6 [pg/ml]	35.0 (3.8 – 775.0)	55.6 (2.0 – 57,050.0)	0.388
CRP [mg/l]	0.2 (0.2 – 51.1)	0.2 (0.2 – 78.2)	**0.031**/*0.271
Hematocrit [%]	46 (10.7 – 59.0)	44 (15.2 – 59.0)	**0.047**/*0.268
Leucocytes [×10^3^/μl]	7.28 (1.98 – 32.00)	7.30 (1.08 – 26.25)	0.889
Thrombocytes [×10^3^/μl]	165 (29 – 235)	160 (15 – 291)	0.258
FII [activity in%]	29.5 (20 – 53)	33 (17 – 65)	0.495/*0.154
FVII [activity in%]	30.5 (13 – 93)	31 (7 – 69)	0.868/*0.330
FX [activity in%]	37.5 (24 – 73)	44 (14 – 89)	0.682/*0.235
ATIII [activity in%]	29 (13 – 51)	29.5 (13 – 48)	0.903/*0.532

*adjusted for gestational age.

Bold numbers are used for results reaching statistical significance.

Allele frequencies for the polymorphisms *F7*-323Ins10 (wt/wt 79.1%, wt/-323Ins10 20.9%) and *VKORC1* -1639G>A (GG 39.1%, GA 46.0%, AA 14.9%) were in Hardy-Weinberg equilibrium and comparable with those reported previously [16,17].

Infants carrying the *F7*-323Ins10 variant tended to have lower FVII levels compared to wildtype infants. The genotype effect on FVII levels became statistically significant after adjustment for gestational age (p = 0.028). Similarly, *F7*-323In10 carriers also showed lower FX levels after adjusting for gestational age. All other clinical and laboratory parameters including occurrence of IVH did not differ between wildtype infants and those carrying at least one *F7*-323Ins10 allele (Table 2).

**Figure 1:**
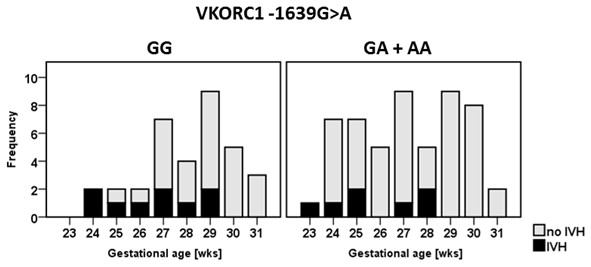
**Distribution of IVH cases according to *VKORC1*-genotype and gestational age.**

Apart from a trend towards lower gestational age, *VKORC1* -1639A carriers revealed higher C-reactive protein (mean 4.8 ± 13.3 mg/l vs. 1.9 ± 8.7 mg/l; p = 0.031) and lower hematocrit levels (mean 47.1 ± 9.8% vs. 43.5 ± 8.8%; p = 0.047) in our cohort (Table 3). The genotype effects on C-reactive protein and hematocrit disappeared after adjustment for gestational age (both p > 0.2). Other laboratory parameters including plasma levels of the vitamin K dependent coagulation factors as determined in the first hour of life did not differ significantly between genotype groups. However, *VKORC* -1639A carriers were less likely to suffer from IVH (GG 26.5%, GA 15.0%, AA 7.7%; p = 0.019 after adjusting for gestational age). Logistic regression analysis including the variables gestational age, 5 minute Apgar score, intubation, incidence of a patent ductus arteriosus, hematocrit, and *VKORC1*-genotype revealed significant contributions of gestational age (OR 0.93 per day (95% CI 0.89-0.98), p = 0.004), and *VKORC1* -1639A carrier status (OR 0.20 (95% CI 0.05-0.80), p = 0.024) to the individual IVH-risk. Figure 1 illustrates the distribution of IVH cases according to *VKORC1*-genotype and gestational age.

## Discussion

In the present study we assessed the impact of two polymorphisms in the vitamin K dependent coagulation system on the coagulation profile and the occurrence of IVH in preterm infants with a gestational age of less than 32 weeks. Consistent with previously published data obtained from adults [18] and one-month-old children [13], carriers of the *F7*-323Ins10 allele showed lower levels of FVII. In adults, a large case–control study comparing 201 patients with spontaneous intracranial hemorrhage and 201 control subjects revealed a 1.54-fold risk for intracranial hemorrhage in carriers of the -323Ins10 allele [19]. The importance of FVII activity for the perinatal coagulation status and morbidity is further demonstrated in *f7*^-/-^ mice who suffer from fatal perinatal bleeding: 70% of them have fatal intra-abdominal bleeding within the first day of life, whereas most of the remaining neonates die from intracranial hemorrhage before the age of 24 days [20]. However, in our cohort we did not observe an association between the *F7*-323Ins10 polymorphism and occurrence of IVH. This is in line with data from a large genotype association study comprising 1009 VLBW infants [7].

In contrast to our previously published data [21], we did not find an association of early postnatal FVII levels and occurrence of IVH in the current cohort. However, apart from a smaller sample size (90 vs. 132 infants) and probably not sufficient statistical power to detect comparatively small effects, these two cohorts are not comparable. Whereas the previously reported cohort comprised exclusively extremely preterm infants with less than 28 weeks of gestational age (n = 132), only 48.9% (n = 38) of the current cohort were born with a gestational age below 28 weeks. Accordingly, total IVH rates differed markedly (43.9% vs. 18.8%), reflecting the close relationship between gestational age and risk to develop IVH. In addition, we and others have demonstrated a significant inverse relationship between factor VII levels and gestational age [15,22,23], and at least in extremely preterm infants with correspondingly immature coagulation profiles, low factor VII levels may represent an independent risk factor for the development of IVH [15].

Interestingly, *F7*-323Ins10 carriers also showed lower FX levels after adjusting for gestational age. Considering the close proximity of the genes encoding the coagulation factors VII and X on chromosome 13q34, linkage of functionally relevant polymorphisms in these two genes may explain this finding.

VKORC1 is considered the key protein of the vitamin K cycle. Its physiological relevance during early stages of life is highlighted by the phenotype of *vkorc1*^*-/-*^ mice who develop normally until birth, but die within 2 to 20 days after birth due to extensive, predominantly intracerebral hemorrhage. The lethal phenotype, which results from severe vitamin K-dependent clotting factor deficiency, can be rescued by oral administration of vitamin K [24]. Similar to *vkorc1*^*-/-*^mice, patients suffering from vitamin K-dependent clotting factor deficiency type 2, an extremely rare autosomal recessive bleeding disorder arising from point mutations in the *VKORC1* gene, also present with severe perinatal intracerebral hemorrhage [25,26]. Considering the decreased enzyme activity and warfarin dose requirement resulting from the relatively frequent *VKORC1* -1639G>A polymorphism, one may speculate that the variant allele (-1639A) might be associated with a higher risk of developing IVH. Conversely, we found a higher IVH risk in *VKORC1*-1639GG homozygotes compared to infants with at least one A-allele. A possible explanation might be the reported alteration of the vitamin K pharmacokinetics mediated by *VKORC1* -1639G>A. In a pilot study comprising five men and five women each per genotype group, *VKORC1*-1639GG homozygotes exhibited a significantly shorter elimination half-time of orally administered vitamin K1 compared to carriers of at least one A-allele [27]. Moreover, in a cohort of 202 adult patients receiving warfarin treatment, the required warfarin dose was significantly reduced in association with decreasing dietary vitamin K intake in *VKORC1*-1639AG heterozygotes. This dietary influence was totally abolished in *VKORC1*-1639AA homozygotes [28]. In another study on 33 over-anticoagulated adult patients, the INR value decreased significantly faster in carriers of the G allele compared to *VKORC1*-1639AA homozygotes [29]. However, the impact of *VKORC1*-1639G>A on vitamin K pharmacokinetics in the pediatric population has, to our knowledge, not been investigated so far. This is of particular clinical importance since preterm infants have a substantially diminished coagulation profile compared to adults [15,22,23] and probably also differ with regard to the pharmacokinetics of vitamin K [30]. Consequently, the higher IVH rate observed in -1639GG infants might be directly linked to genotype effects on vitamin K metabolism and related short term changes in the coagulation profile following the routinely administered vitamin K prophylaxis on the first day of life.

In the present cohort, levels of the vitamin K dependent clotting factors did not differ according to the *VKORC1* -1639G>A genotype. However, it is important to note that all laboratory parameters were assessed in the first hour of life. Presuming that pharmacokinetics of vitamin K depend on *VKORC1* -1639G>A, plasma levels of the vitamin K dependent clotting factors might develop differently in the course of the following hours or days, and consequently may affect the individual risk to develop IVH. Further research is required to assess the impact of the *VKORC1* -1639G>A polymorphism on vitamin K pharmacokinetics and the coagulation profile in extremely preterm infants with a high risk to develop IVH. In this context, it seems advisable to re-evaluate dose, interval and route of the routine vitamin K administration in this particular group of neonates.

## Conclusion

In the present study, we assessed the impact of functional polymorphisms in the *F7* and *VKORC1* genes on the coagulation profile and the risk to develop IVH in a cohort of preterm infants. Our data support the assumption that genetic variants in the vitamin K-dependent coagulation system influence the coagulation profile and the IVH risk in this particular group of neonates with an increased risk of developing IVH. Further studies focussing on vitamin K kinetics and short-term changes in the coagulation profile, particularly during the first days of life, are required to further understand a possible link between IVH risk and genetic variants affecting the metabolism of vitamin K.

## Abbreviations

CPAP: Continuous positive airway pressure; CRIB: Clinical risk index for babies; F7: Coagulation factor 7; Insure: *In*tubate, *sur*factant, *e*xtubate; IVH: Intraventricular hemorrhage; NICU: Neonatal intensive care unit; SGA: Small for gestational age; SNP: Single nucleotide polymorphism; VKORC1: Vitamin K epoxide reductase complex 1; VLBW: Very low birth weight.

## Competing interests

The authors declare that they have no competing interests.

## Authors’ contributions

CS was responsible for medical care of the patients, performed genotyping, analyzed the data and drafted the initial manuscript. SS collected clinical data, performed genotyping and drafted the initial manuscript. MW, HJH and JO designed the study, supervised coagulation tests and genotyping. FS supervised genotyping and analyzed the data. PB and AH were responsible for medical care of the patients and designed the study. All authors reviewed and approved the final manuscript.

## Pre-publication history

The pre-publication history for this paper can be accessed here:

http://www.biomedcentral.com/1471-2431/14/219/prepub
